# Nitrogen Fixation by *Trichodesmium* and unicellular diazotrophs in the northern South China Sea and the Kuroshio in summer

**DOI:** 10.1038/s41598-018-20743-0

**Published:** 2018-02-05

**Authors:** Chao Wu, Fei-Xue Fu, Jun Sun, Satheeswaran Thangaraj, Laxman Pujari

**Affiliations:** 1 Institute of Marine Science and Technology, Shandong University, Jinan, 250110 China; 2 Department of Biological Sciences, University of Southern California, Los Angeles, California, 90089 USA; 3 College of Marine and Environmental Sciences, Tianjin University of Science and Technology, Tianjin, 300457 China; 4 Tianjin Key Laboratory of Marine Resources and Chemistry, Tianjin University of Science and Technology, Tianjin, 300457 China

## Abstract

Distribution of diazotrophs and their nitrogen fixation activity were investigated in the northern South China Sea (nSCS) and the Kuroshio from July 16th to September 1st, 2009. N_2_ fixation activities in whole seawater and <10 μm fraction at the surface were measured by acetylene reduction assay. Higher activities were observed at the East China Sea (ECS) Kuroshio and the nSCS shelf. The nSCS basin showed a low N_2_ fixation activity. The <10 μm fractions (unicellular diazotrophs) contributed major portion to the whole-water activity in the survey time, indicating that nanoplanktonic cyanobacterias were the major diazotrophs in the survey area. Daily N_2_ fixation rates of *Trichodesmium* ranged from 0.11 to 9.83 pmolNtrichome^−1^ d^−1^ with an average of 4.03 pmolNtrichome^−1^ d^−1^. The Luzon Strait and the ECS Kuroshio had higher N_2_ fixation rates of *Trichodesmium* than the nSCS shelf and basin. Calculated activities of *Trichodesmium* at most stations were moderately low compared with that of the whole-water. The contribution of N_2_ fixation by the whole-water to primary production ranged from 1.7% to 18.5%. The estimated amount of new nitrogen introduced by *Trichodesmium* contributed up to 0.14% of the total primary production and 0.41% of the new production in the Luzon Strait.

## Introduction

N_2_ fixation is an important process in adding “new” nitrogen to oligotrophic ocean ecosystems^1^. The current focus in assessing the global role of the upper ocean in sequestering atmospheric CO_2_ has elevated the importance of quantifying marine N_2_ fixation^2^. The filamentous non-heterocystous cyanobacteria *Trichodesmium* and diatom-diazotroph associations containing the cyanobiont *Richelia intracellularis* were traditionally considered to be the most important contributors to open-ocean N_2_ fixation. *Trichodesmium* mainly inhabits stratified, oligotrophic, tropical and subtropical oceans which are characterized by low nutrient concentrations, clear waters, and deep light penetration^3–5^. N_2_ fixation from *Trichodesmium* may supply up to half of the N required to sustain the rate of the annual particulate N (PN) export from the euphotic zone at Sta. ALOHA (22°45′N, 158°00′W) in the subtropical North Pacific Ocean^6^. However, the discovery of two unicellular diazotrophic cyanobacteria [UCYN group A (UCYN-A) and *Crocosphaera watsonii* (group B)], whose abundances and N_2_ fixation rates can be equal to or greater than those of *Trichodesmium*, demands the scientists to make a reassessment of the N inputs to the global ocean via N_2_ fixation^7–9^.

The South China Sea (SCS) is the second largest marginal sea in the world. It is located in the tropical-subtropical western North Pacific and has a surface area of about 3.5 × 10^6^ km^2^. Because of the effects of the monsoons and eddies, the northern SCS (nSCS) displays a complex hydrological and biological characters. The surface circulation of the nSCS changes seasonally in response to the prevalent monsoons. Overall seasonal circulation in the SCS is cyclonic in winter and anti-cyclonic in summer^10^. Upwellings and downwellings are forced by the interaction of monsoons and eddies^11^. The nSCS exhibits strong variations of temperature and salinity from coastal region to the open sea. The surface water of the nSCS and the Kuroshio is generally warm, stratified and oligotrophic which is supposedly an ideal habitat for N-fixers especially in summer^4^. The Kuroshio and nSCS are two neighboring and interacting waterbodies. The Kuroshio spans a wide range of latitudes from tropical 15°N to temperate 40°N. The upstream Kuroshio intrudes into the SCS through the Luzon Strait which is the deepest passage between the western Pacific and the SCS. It is suggested that the Kuroshio has a branch intruding into the nSCS in winter, but it sometimes intrudes into the nSCS in summer mostly in the form of “loop” and “extend”^12,13^. After passing southeastern Taiwan, the Kuroshio water flows northward and exchange their energy and matter with the East China Sea. The Kuroshio and the nSCS show different nutrient dynamics. Gong *et al*.^14^ observed that nutricline in the northern SCS is shallower than the Kuroshio^14^. Chen *et al*.^15^ also observed that in summer nitracline is shallower in the SCS than in the Kuroshio^15^.

Most published studies of marine nitrogen fixation were focused on the North Pacific^8,16–19^ and North Atlantic^20–23^. Compared with previous studies in the East China Sea^24^, the SCS^4,15,25^ and the Kruoshio^26^ have been studied deficiently. The present study was conducted to investigate the horizontal and vertical variation of *Trichodesmium* abundance and it’s *in situ* nitrogen fixation rates in the nSCS and the Kuroshio. Size fractionation experiments were also conducted to compare the relative importance of the activity between different size classes of diazotrophs in the Kuroshio and nSCS. In addition, environmental factors were measured to determine the main ecological factors that control the nitrogen fixation.

## Results

### Hydrographic conditions

**Figure 1:**
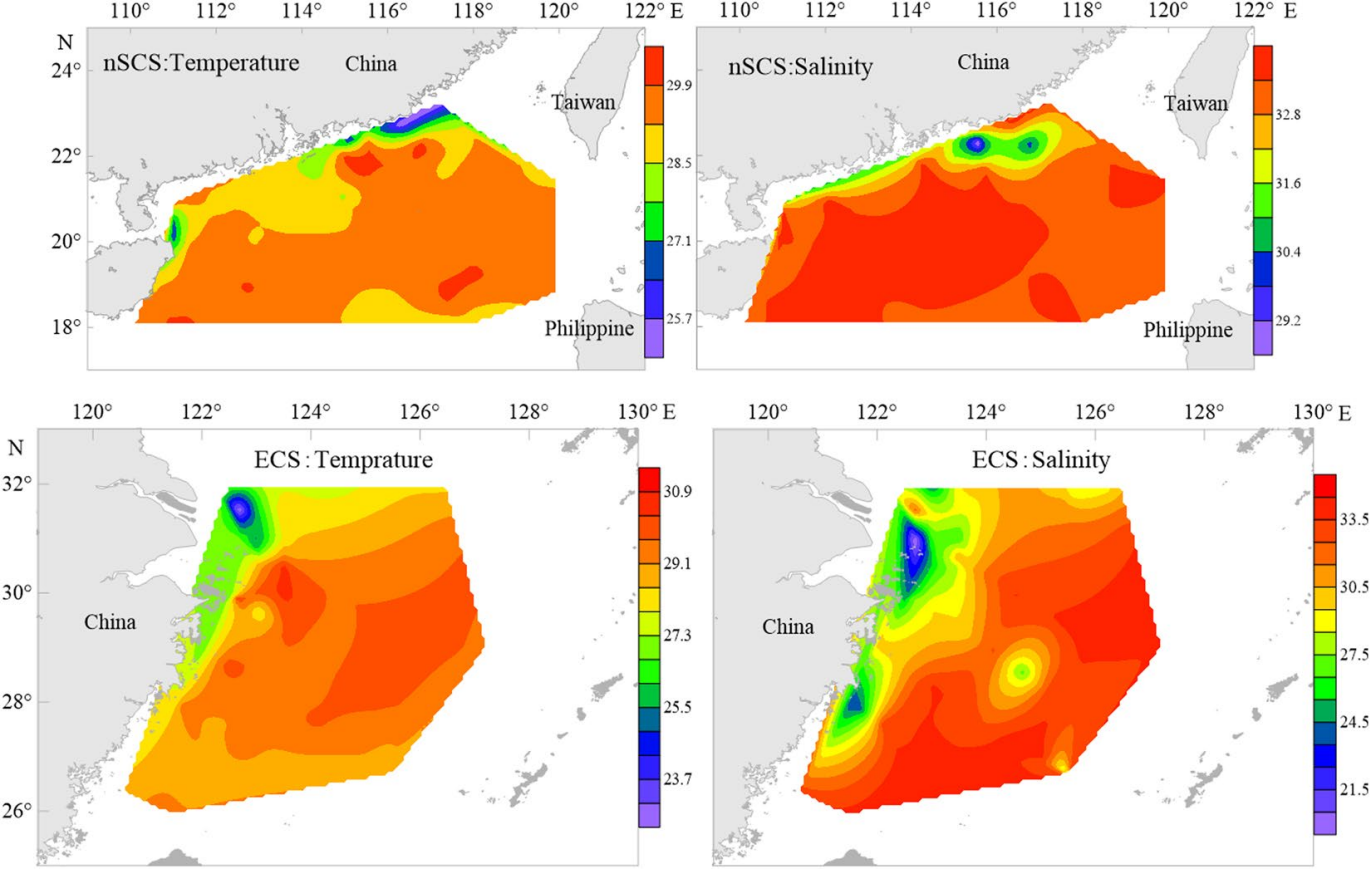
Surface sea temperature and salinity in the northern SCS and East China Sea. Note for software: Golden Software Surfer 11 (http://www.goldensoftware.com/).

In nSCS, sea surface temperature (SST) ranged between 25.3 °C and 30.5 °C with an average of 29.3 ± 0.8 °C. Surface sea salinity ranged from 28.4 to 33.8 with an average of 33.2 ± 0.7. Most regions in nSCS were characterized by high temperature and high salinity (Fig. 1). In East China Sea, regions that heavily influenced by the Kuroshio also had high temperature and high salinity (Fig. 1). In nSCS during survey time, surface NO_3_^−^ + NO_2_^−^, SRP, and silicate concentrations were lower than 0.15 μmol L^−1^, 0.1 μmol L^−1^, and 3 μmol L^−1^ respectively. The nutrient concentrations of most stations at the survey region were undetectable, especially at the Luzon Strait. In the East China Sea Kuroshio region, surface NO_3_^−^ + NO_2_^−^, SRP, and silicate concentrations were also very low or undetectable.

### Horizontal distribution of the N2 fixation activity

**Figure 2:**
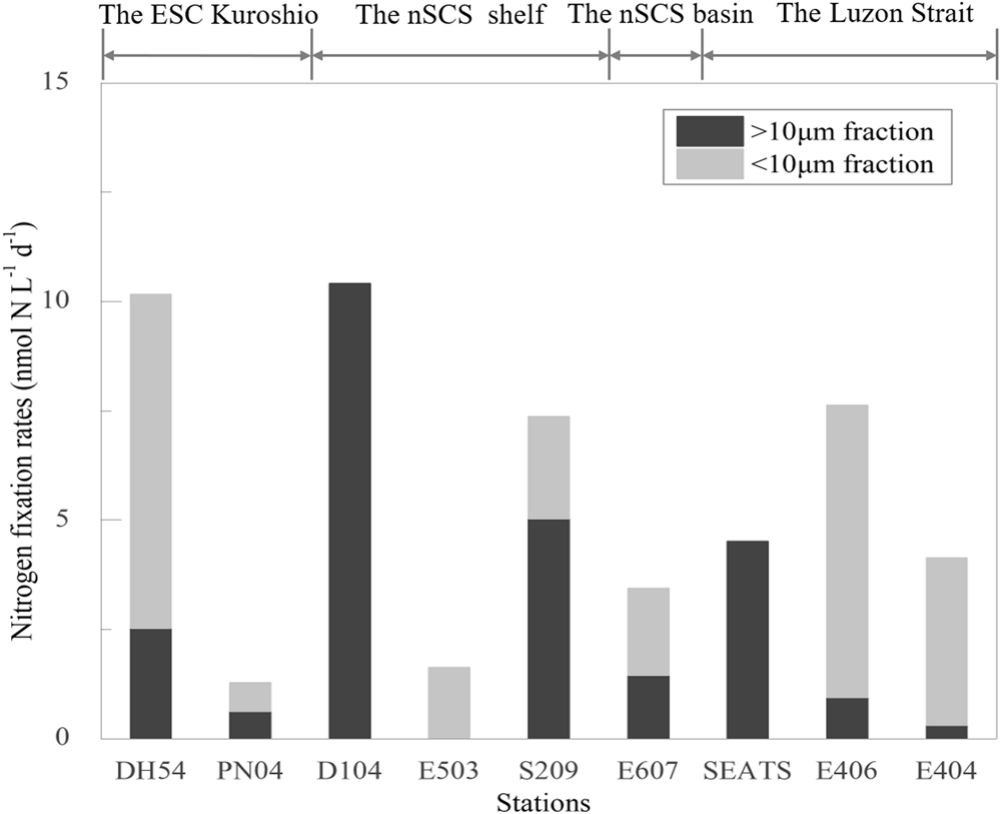
Distributions of N_2_ fixation activity of the whole-water sample (open circle) and <10 μm fractions (solid circle) at the surface. Note for software: Origin 8.5 PRO (http://www.originlab.com/).

N_2_ fixation rate of whole-water at the surface ranged from 1.14 to 10.40 nmol N L^−1^d^−1^ with an average of 4.89 nmol N L^−1^d^−1^. N_2_ fixation rate of the <10 μm fraction at the surface ranged from 0.63 to 7.61 nmol N L^−1^d^−1^ with an average of 3.67 nmol N L^−1^d^−1^. N_2_ fixation rate of whole-water changed a lot among different survey stations. High N_2_ fixation of whole-water at the surface was observed in the ECS Kuroshio and the nSCS shelf. The nSCS basin showed low N_2_ fixation activity. These rates were in the same order of magnitude as those measured under non-bloom conditions in the East China Sea, the southern Yellow Sea and the nSCS coastal upwelling^27,28^. The high N_2_ fixation rates in the nSCS shelf were probably attributed to diazotrophic blooms in sampling stations. N_2_ fixation activity in the <10 μm fraction exhibited a similar distribution to that of the whole-water in survey region (Fig. 2). Our experiments showed that unicellular diazotrophs contributed significantly to N_2_ fixation in both the SCS and the Kuroshio.

### Vertical distribution of the N2 fixation activity

**Figure 3:**
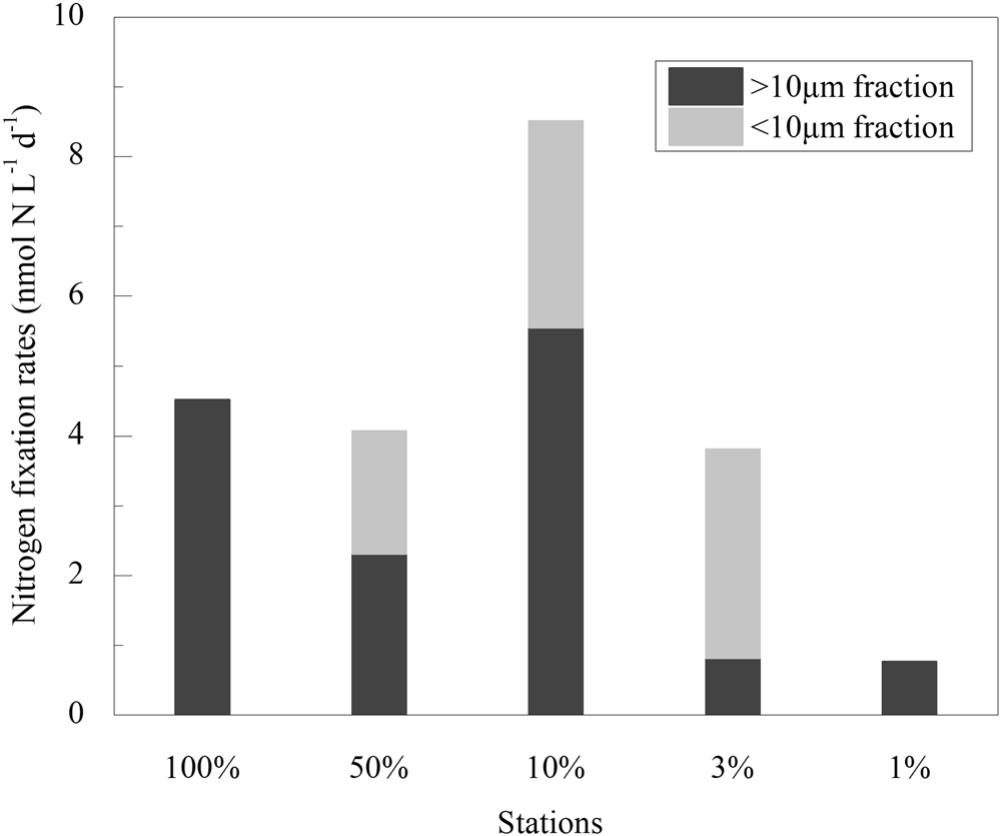
Vertical distribution of N_2_ fixation rate at Sta. SEATS. Note for software: Origin 8.5 PRO (http://www.originlab.com/).

The whole-water N_2_ fixation rate at Sta. SEATS ranged from 0.76 to 8.50 nmolNL^−1^d^−1^ and the average was 4.33 nmolNL^−1^d^−1^ (Fig. 3). The >10 μm fractions N_2_ fixation rates decreased with the light intensity except for the 10% light depth, while the <10 μm fractions N_2_ fixation rates increased with the light intensity in the middle three light depth. Light intensity is an important factor that determines the distribution of diazotrophs in the water column. *Trichodesmium* usually prefers the top sunlight of 75 m, but unicellular cyanobacteria tend to appear at deeper layers and down up to ~150 m^29^. The <10 μm fractions N_2_ fixation rate of 100% and 1% light depth were undetectable in our study. The rare occurrence of unicellular cyanobacteria at the SEATS station has been verified by molecular study^30^. The growth of unicellular cyanobacteria in this station could be limited by iron resources due to the competition from non-diazotrophs. The maximum whole-water N_2_ fixation rate was observed in the 10% light depths in our study, and the result was different from the previous study that the maximum N_2_ fixation was in the surface layer and decreasing with depth in the lower euphotic zone^31^. Notably, the main factor resulted in this situation was the N_2_ fixation rate by >10 μm fractions in the 10% light depths were larger than other depths extraordinarily. The same phenomenon was also observed in the nSCS^15^ and North Pacific^32^. A possible reason generated for this phenomenon probably due to the result of the downward flux of *Trichodesmium* spp^33^.

### *Trichodesmium* fixation rate

**Figure 4:**
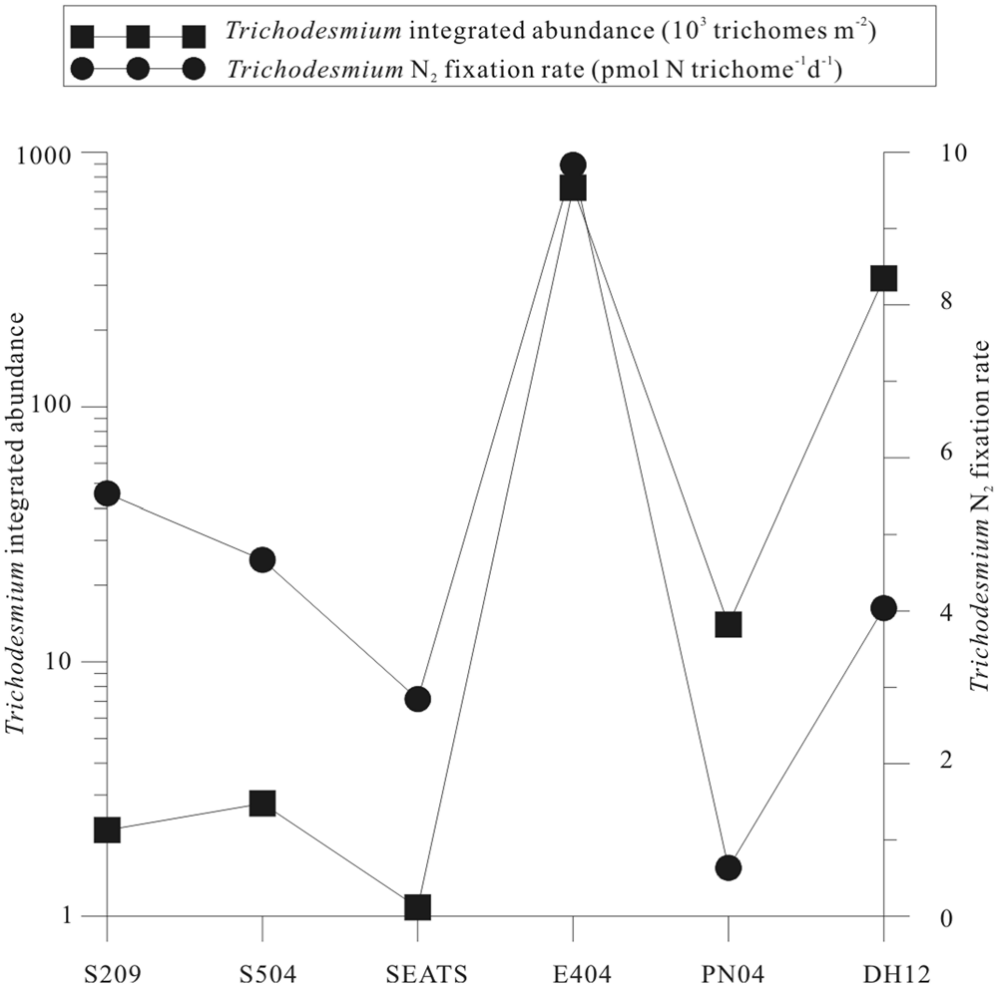
*Trichodesmium* integrated concentrations and daily N_2_ fixation rate in survey region. Note for software: Origin 8.5 PRO (http://www.originlab.com/).

Hourly N_2_ fixation rates of *Trichodesmium* were converted to daily rates with day length, assuming that *Trichodesmium* fixed N_2_ only during the daytime^34^. Daily N_2_ fixation rates of *Trichodesmium* ranged from 0.11 to 9.83 pmolNtrichome^−1^ d^−1^ with an average of 4.03 pmolNtrichome^−1^ d^−1^. The maximum rate of 9.83 pmolNtrichome^−1^ d^−1^ was recorded in Sta. E404 in the Luzon Strait for an experiment containing longitudinal colonies (cultivated from 8:00 am to 2:00 pm). The second maximum rate of 9.64 pmolNtrichome^−1^ d^−1^ was also recorded in Sta. E404 for an experiment containing radial colonies (cultivated from 8:00 to 10:00 am). In general, the Luzon Strait and ECS Kurosio had higher N_2_ fixation rates by *Trichodesmium* than the nSCS shelf and basin. The variations of N_2_ fixation rates by *Trichodesmium* were consistent with that of the *Trichodesmium* abundances (Fig. 4). In the ECS Kuroshio, Sta. PN04 showed a low N_2_ fixation rate by *Trichodesmium* because the experiment was conducted at night (11:00 pm to 1:00 am of the next day).

**Figure 5:**
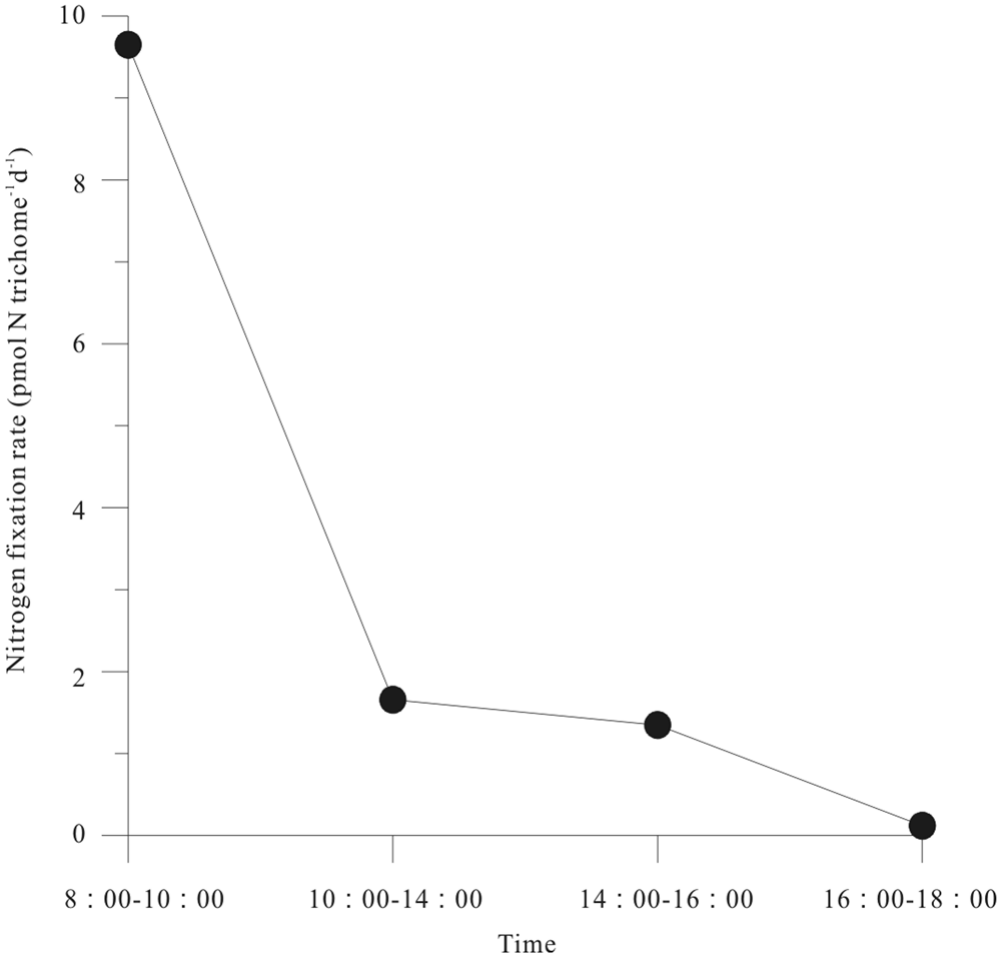
Daily variations of N_2_ fixation rate by *Trichodesmium* at Sta. E404. Note for software: Origin 8.5 PRO (http://www.originlab.com/).

A time-series assay was also conducted at Sta. E404. N_2_ fixation rates by *Trichodesmium* radial colonies were measured at 8:00–10:00, 10:00–14:00, 14:00–16:00 and 16:00–18:00. The maximum N_2_ fixation rate occurred in the 8:00 to 10:00 am. The N_2_ fixation rate by *Trichodesmium* decreased sharply from 8:00 am to 6:00 pm, and the rate dropped off rapidly to near zero prior to the onset of the dark period. The N_2_ fixation rate during 4:00 to 6:00 pm was almost undetectable (Fig. 5). The results generated from field incubation were consistent with previous study that high N_2_ fixation rates were measured for ~6 hours surrounding the middle of the photoperiod and N_2_ fixation declined sharply in the latter light period^35^.

### *Trichodesmium* standing crop and their contribution to total Chl *a*

In most instances, the individual free trichomes were the absolute dominant population in global ocean^4^. These free trichomes could form macroscopic colonies or aggregates which known as ‘puffs’ and ‘tuffs’ in extreme oligotrophic conditions^36^. In the present study, three living forms of *Trichodesmium* were observed during the survey time: the free trichome, the radial colony and the longitudinal colony. Likewise, the primary form of *Trichodesmium* in most stations were free living trichomes rather than colonies. This trait was in better agreement with previous study of *Trichodesmium* in the SCS and southern ECS^4,37^.

**Figure 6:**
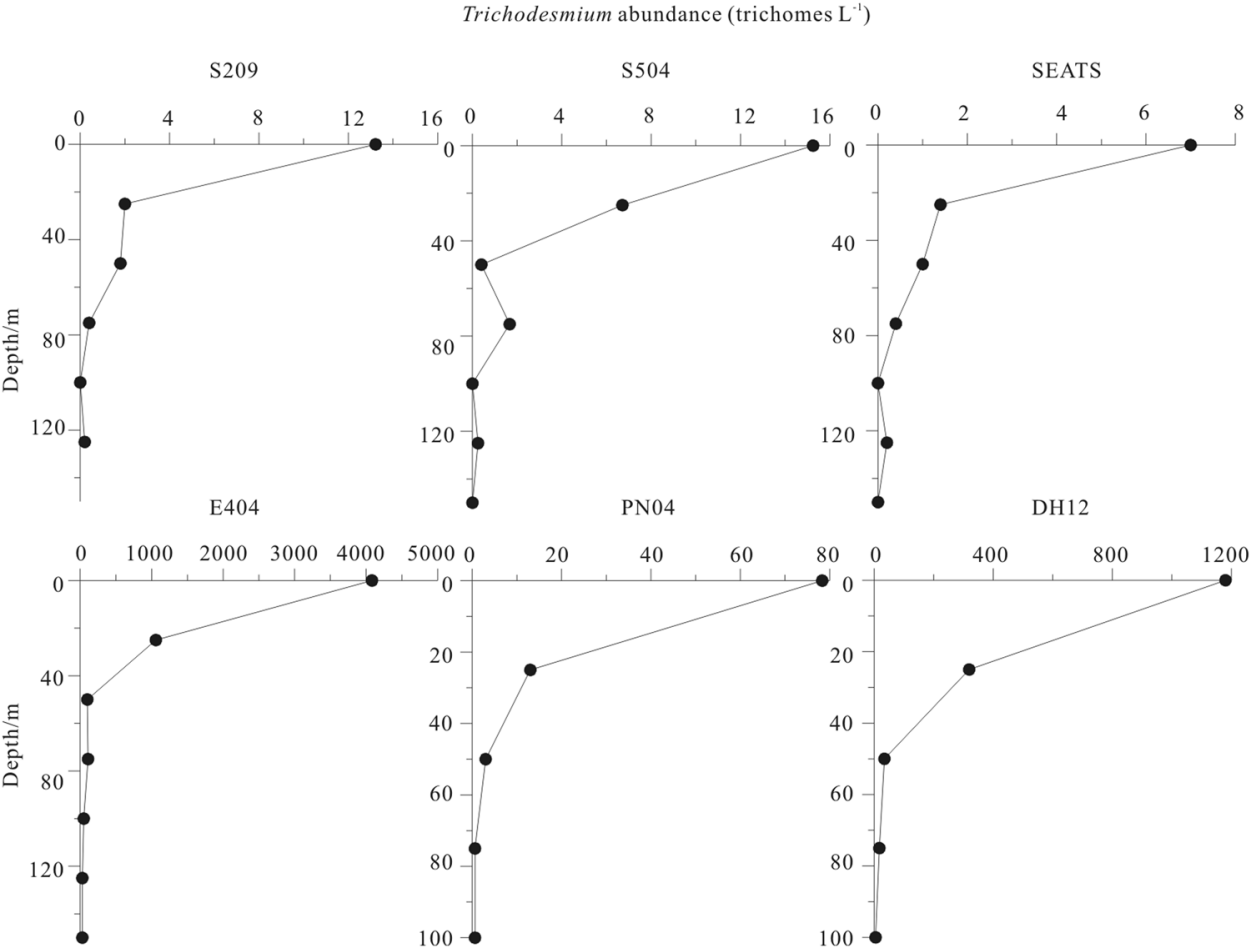
Vertical distribution of *Trichodesmium*. Note for software: Origin 8.5 PRO (http://www.originlab.com/).

The average number of trichomes per radial colony and longitudinal colony in all stations were 80 and 24, respectively. The average number of cells per trichome was 60. This data was typically higher than previous reports in the SCS that colonial forms of *Trichodesmium* were usually composed of less than 10 trichomes per colony^37^. Our values were nevertheless lower than other oceans such as tropical North Atlantic Ocean (mean = 98 ± 11, range = 5–153 spp./colony), station ALOHA in subtropical eastern North Pacific Ocean (mean = 182, range = 10–375 spp./colony) and subtropical eastern North Atlantic gyre (mean = 112 ± 47 for puffs and 49 ± 16 for tufts, range = 57–156 for puffs and 31–63 for tufts spp./colony)^38–40^. *Trichodesmium* integrated concentrations ranged from 2.18 to 726.17 × 103 trichomes m^−2^ and the average was 177.65 × 103 trichomes m^−2^. The Luzon Strait and the ECS Kuroshio had higher *Trichodesmium* abundances than the nSCS shelf and basin. Vertical distribution of *Trichodesmium* showed that from the surface to the bottom the abundances of *Trichodesmium* decreased dramatically (Figs 5 and 6).

**Figure 7:**
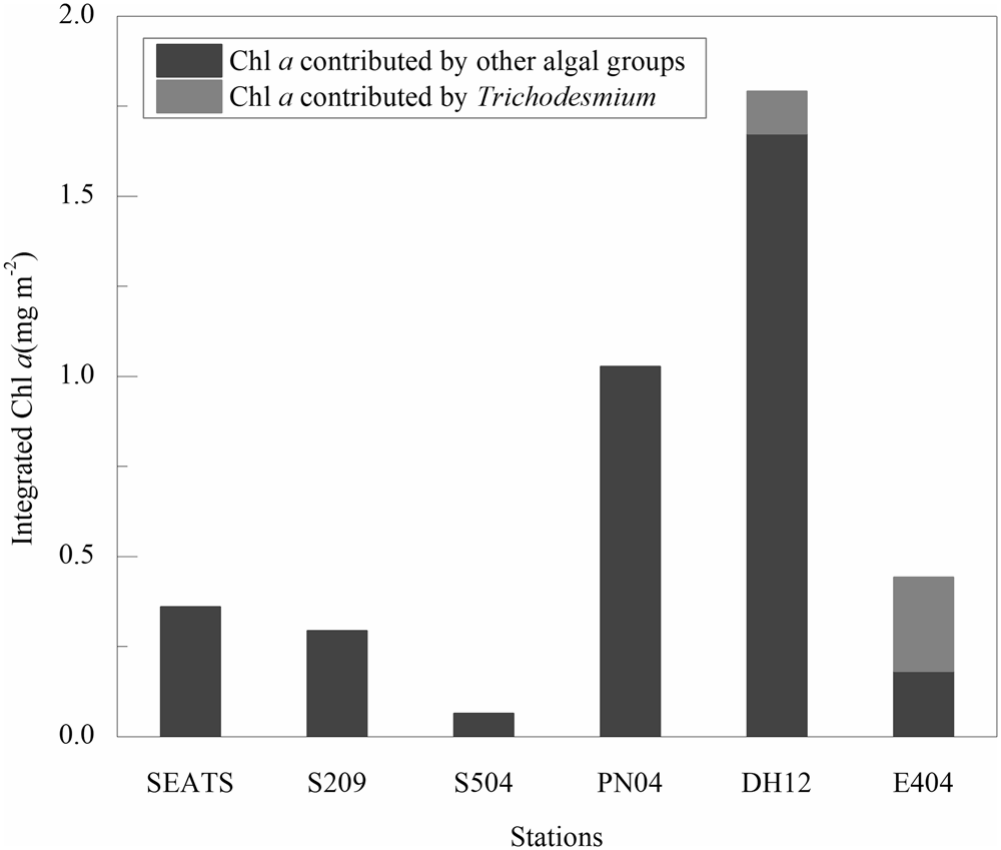
Chl *a* concentration contributed by *Trichodesmium*. Note for software: Origin 8.5 PRO (http://www.originlab.com/).

According to the *Trichodesmium* integrated abundances and Chl *a* concentration per trichome measured at the sampling stations, estimated Chl *a* contributed by *Trichodesmium* varied from 0.1% to 58.5% of the total Chl *a* (Fig. 7). The values in the ECS Kuroshio and the Luzon Strait were significantly higher than that in the nSCS. Likewise, obvious spatial difference was also observed in another ocean. For example, literature have reported that *Trichodesmium* to account for, on average, about 61%, 18% and 5% of total Chl *a* in the Caribbean Sea, the eastern North Pacific Ocean and the Sargasso Sea, respectively^39,41^. The difference of the contribution in different regions might be caused by the variation of their dominant phytoplankton^5^.

### Comparison of N_2_ fixation rate of *Trichodesmium* and other diazotrophs and their contributions to the primary production

Calculated activities of *Trichodesmium* at most stations were quite low in the whole-water (<0.1 nmolNL^−1^d^−1^) except at Sta. E404 where the N_2_ fixation rate by *Trichodesmium* was as high as 57.7 nmolNL^−1^d^−1^. On the contrary, unicellular diazotrophs were accounted for a great part of the whole-water activity at most stations in the survey time. N_2_ fixation was converted to carbon production using the Redfield ratio (C:N = 6.6:1) for comparison with the primary production. N_2_ fixation by the whole-water was calculated to contribute to 1.7% to 18.5% of the primary production (Table 1). According to the *Trichodesmium* integrated abundances and nitrogen fixation rates per trichome observed at the sampling stations, estimated integrated nitrogen fixation rates from *Trichodesmium* varied from 0.0031 to 7.0 μmol N m^−2^ d^−1^. Calculated N_2_ fixation rate by *Trichodesmium* showed it could contribute up to 0.14% of the primary production (Table 2).

**Table 1:** Estimated whole-water N2 fixation rate and its contribution to primary production at surface water.

Station	N_2_ fixation rate (μmolNm^−3^d^–1^)	Primary production (mgCm^−3^d^−1^)	Contributions of N_2_ fixation to primary production (%)
DH27b	1.14	5.24	1.7
S209	7.36	4.18	13.9
E607	3.43	1.47	18.5
SEATS	4.50	9.44	3.8
LE09	2.22	7.30	2.4
E404	4.12	8.73	3.7

**Table 2:** Calculated *Trichodesmium* N_2_ fixation rate and its contribution to the primary production.

Station	N_2_ fixation rate (μmolNm^−2^d^−1^)	Primary production (mgCm^−2^d^−1^)	Contributions of N_2_ fixation to primary production (%)
S504	0.013	94.2	0.0011
E404	7.0	381.7	0.14
S209	0.012	242.7	<0.001
SEATS	0.0031	445.1	<0.001
DH12	1.3	400.3	0.025

## Discussion

In the summer, most regions of the nSCS have high SST except some regions which are influenced by the upstream. The stratified water of the nSCS results in the low supplement of the nutrient from deeper water. Originating from the southeast of Taiwan and east of the Luzon Strait, the Kuroshio is characterized by high temperature, high salinity, high transparency and low nutrients concentrations. As a result, most regions of the nSCS and the ECS Kuroshio were warm, stratified and oligotrophic which favored the growth of the diazotrophs. As an important diazotraph in the oligotrophic ocean and the Kuroshio water species indicator, *Trichodesmium*’s distribution and nitrogen fixation rate in the nSCS and neighboring Kuroshio play an important role in the biological production, especially the new production of the SCS and the Kuroshio. Although conditions in both the SCS and the Kuroshio seem favorable for *Trichodesmium* growth, the Kuroshio had a higher abundance of *Trichodesmium* and nitrogen fixation rate than the nSCS. The standing crop and nitrogen fixation rate of *Trichodesmium* in the SCS basin is very low (Fig. 4 and Table 2). Shiozaki *et al*.^26^ suggested the high abundance of *Trichodesmium* in the ESC Kuroshio were ascribable to the supply of *Trichodesmium* spp. and other diazotrophs from the surrounding islands^26^. The Kuroshio is a western boundary current which originating from the southeast of Taiwan and east of the Luzon Strait, and enters the East China Sea (ECS) northeast of Taiwan. During our investigations, the surface abundance of *Trichodesmium* in Sta.E404 in the Luzon Strait was two orders of magnitude higher than stations in the nSCS and several times higher than stations in the ESC Kuroshio (Fig. 6). These results proved *Trichodesmium* spp. would likely to be transported to the mainstream of the Kuroshio from the Luzon Strait.

*Trichodesmium* spp. were traditionally perceived to be the most abundant cyanobacteria in the marine, and it has been demonstrated to contribute the vast majority of N_2_ fixation rates and primary production to the ocean^3,42^. However, increasing studies have shown that unicellular diazotrophs often appear to be of equal or greater abundance to the other known N_2_ fixers due to the revolution use of polymerase chain reaction (PCR) techniques^7,31^. A DIC budget analysis at the SEATS in the SCS basin demonstrated that unicellular diazotrophs contributed enormously to the N_2_ fixation, which were thus an indispensable part of the N cycle study in the SCS^43^. Chen *et al*.^31^ observed that unicellular diazotrophs contributed 65% and 50% of the total N_2_ fixation in the SCS and the Kuroshio, respectively^31^. According to the results of size fractionation experiments in the present study, unicellular diazotrophs were important N_2_ fixers in the surface of the nSCS and the Kuroshio. However, investigation on the distribution of the unicellular diazotrophs and their phylogenetic relationships with the unicellular diazotrophs from the tropical North Atlantic and Pacific oceans remain insufficient. Similar situation happens to *Richelia intracellularis*. Further studies of the other diazotrophs including their distribution and nitrogen fixation rate are needed in order to estimate the role of the N_2_ fixation in the nutrient and carbon cycling in SCS and the Kuroshio.

In the open ocean, many factors can influence the activity and diversity of diazotrophs. Water temperature is a major factor controlling the distribution of diazotrophs^44^. *Trichodesmium* is distributed where temperatures are between 20 °C and 30 °C and thrives when temperature is 25 °C or warmer^45,46^. By comparison, unicellular diazotrophs often present in deeper and colder waters than *Trichodesmium*^29^. In the present study, however, the correlation between size-fractioned N_2_ fixation and temperature was not significant in both the nSCS and the Kuroshio. This might be attributed to the unusually strong wind and highly turbulent surface during summer cruise^31^. Diazotrophs, especially *Trichodesmium*, usually thrives in relatively stable environment. Previous works had demonstrated the importance of wind as a driving force for promoting the growth of *Trichodesmium* and diatoms^47,48^. Generally it has been found that highest concentrations of *Trichodesmium* occur during prolonged calm wind (<10 knots ≈ 5.14 m/s) conditions whereas the highest diatom concentrations occur during windy (>15 knots ≈ 7.72 m/s) conditions. Calm waters with low turbulence benefit bundle formation and tend to enhance N_2_ fixation^4^. During the survey time, influenced by the typhoon “Swan” and “Morakot”, a much lower abundance of *Trichodesmium* was detected at Sta. SEATS (1.08 × 10^3^ trichomes m^−2^) compared with the previous study^4^. Besides, diazotrophs growth rates are presumably limited by the availability of non-N nutrients, iron and phosphate are key factors limiting diazotrophs N_2_ fixation and growth rates^49^. Iron is a important cofactor of nitrogenase, and it plays an crucial role in the synthesis and expression of nitrogenase in diazotroiphs^50^. According to literatures, dissolved Fe (dFe) in surface waters ranged from 0.2–0.3 nmol L^−1^ in the SCS basin stations and 1.8–43.2 nmol L^−1^ in the northwest SCS coastal stations^28,37^. It was suggested that Fe availability may limit the rates of N_2_ fixation in the SCS basin, while it was not happened in the nSCS coastal stations. As for the phosphate, it is a major component of cells which could also limit the growth of diazotroiphs^29,51^. In the present study, the phosphate (P) concentration in most of the areas was very low or undetectable. However, both *Trichodesmium* and unicellular diazotrophs had form a number of mechanisms to overcome P limitation. A key characteristic of *Trichodesmium* is the presence of gas vesicles, which could provide buoyancy and help them migrate vertically in the water column to optimize light intensity or to obtain P and other nutrients from deep waters^3^. Unlike with *Trichodesmium*, unicellular diazotrophs could not migrate vertically in the water column, but they had been proved to own a robust capacity for scavenging phosphorus in oligotrophic systems due to the presence of high-affinity phosphate scavenging systems^52^.

In addition, nitrogenase is instantaneously and irreversibly inactivated by oxygen, N_2_ fixation is therefore a strictly anaerobic process^53^. However, many studies have demonstrated that both natural populations and laboratory cultures of *Trichodesmium* could fix N_2_ exclusively in the daylight hours when photosynthetic activity takes place simultaneously^54,55^. Our result in Sta. PN04 and Sta. E404 also proved that *Trichodesmium* presented to be a daytime N_2_ fixer. There are several hypotheses which aims to explain this phenomenon, but it is still an enigma how *Trichodesmium* can evolve O_2_ while simultaneously fixing N_2_. The most popular hypothesis is that *Trichodesmium* can use a combination of spatial and temporal separation strategy of N_2_ fixation and photosynthesis within the photoperiod^35,42,55^. Staal *et al*.^55^ pointed that light stimulation of nitrogenase activity was most occurred at low O_2_ concentration, while light stimulation became gradually less important at high O_2_ concentration^55^. This result suggested the reduction of N_2_ fixation in the latter stages of the light period was primarily attributed to the high net production of O_2_ in the latter stages. Furthermore, nitrogenase activity can be recovered after a dark incubation when the cells were subsequently incubated in the light. Similarly, low O_2_ concentration is in favor of the recovery of nitrogenase activity^55^.

Our results were comparable with global ocean, and higher than other study in the same area which might be caused by the discrepancy between the two methods (Table 3). On the basis of a C: N = 6.6, N_2_ fixation by the whole-water was calculated to contribute to 1.7% to 18.5% of the primary production in surface waters, suggesting that N_2_ fixation plays a very important role in supporting surface phytoplankton N demand in some stations. Such values were exceeded the previous studies in the nSCS, and were comparable with N input by biological N_2_ fixation in other oceans^27,28,56^. The variation of N_2_ fixation rate by *Trichodesmium* was consistent with that of the standing crop of *Trichodesmium*. According to the result of Chen *et al*.^4^ new production from *Trichodesmium* N_2_ fixation was higher in the Kuroshio than the nSCS in summer, we also found a higher N_2_ fixation rate by *Trichodesmium* in the Kuroshio (Table 2)^4^. Chen *et al*.^4^ found the ratios of N_2_-fixation-based new production by *Trichodesmium* to NO_3_-N plus N_2_-fix were usually <0.1^4^. Based on *f*-ratio 0.34 at the Kuroshio and 0.22 at nSCS reported by Chen *et al*.^4^, *Trichodesmium* could contributed up to 0.4% of the new primary production at the Luzon Strait and <0.1% of the new primary production at the nSCS basin^4^. Compared with the previous studies, we got lower N_2_ fixation rate and abundance of *Trichodesmium* (Table 4).

**Table 3:** Surface N_2_ fixation rates and dominant diazotroph (s) of different Oceanic Region. ARA = acetylene reduction assay.

Location	Surface N_2_ fixation rate (nmol N L^−1^d^−1^)	Method	Dominant Diazotroph (s)	Reference
nSCS and ECS Kuroshio	1.14–10.40	ARA	Unicellular diazotrophs	This study
nSCS coastal upwelling	0.1–5.6	^15^N_2_ gas bubble	Unknow	^28^
SCS (Mekong River plume)	0.59–22.77	^15^N_2_ gas bubble	*Trichodesmium* spp. and DDAs	^63^
Western North Pacific	≈0.3–22	ARA	Unicellular diazotrophs	^64^
North Pacific	0.360–3.05	^15^N_2_ gas bubble	Heterotrophic bacteria	^65^
Northeast Atlantic	<0.4	ARA	Unicellular diazotrophs	^66^
Eastern North Atlantic	0–151.2	^15^N_2_ gas bubble	*Trichodesmium* and UCYN-A	^67^
Arabian Sea	0.8–225	^15^N_2_ gas dissolution	*Trichodesmium* bloom	^68^
Indian Ocean	0.18–1.27	^15^N_2_ gas bubble	Heterotrophic bacteria	^69^

**Table 4:** Calculated *Trichodesmium* N_2_ fixation rate in the South China Sea and the Kuroshio with literature reports of estimated N_2_ fixation in the adjacent area.

Location	N_2_ fixation rate (μmolNm^−3^d^−1^)	Method	Primary production (mgCm^−2^d^−1^)	Nitrate-uptake-based new production	Reference
Southeastern ECS	126	ARA			^70^
Kuroshio	55				^70,71^
ECS (*Trichodesmium erythraeum*)	279	ARA			^71^
ECS (*Trichodesmium thiebautii*)	685				
Kuroshio winter	2.4	^15^N_2_ gas bubble	530	270	^4^
Kuroshio spring	34.7		540	140	
Kuroshio summer	168.1		510	160	
SCS winter	1.2		620	230	
SCS spring	7.3		500	210	
SCS summer	12.6		360	80	
SCS autumn	5.0		540	250	
ECS Kuroshio	1.29	ARA	400.3		This study
Luzon Strait	7.01		381.7		
SCS basin	0.0031		445.1		
SCS shelf	0.012		242.7		

ARA = acetylene reduction assay.

## Conclusion

In conclusion, the abundance of *Trichodesmium* were higher in the Luzon Strait and ECS Kuroshio than the nSCS shelf and basin, and so does the N_2_ fixation by *Trichodesmium*. It was suggested that the high abundance of *Trichodesmium* in the ECS Kuroshio were ascribable to the supply of *Trichodesmium* spp. and other diazotrophs from the surrounding islands. Nevertheless, it is still poorly understood and additional research is needed to resolve this question. According to the size-fractioned N_2_ fixation rates, unicellular diazotrophs also contributed significantly to N_2_ fixation in both the SCS and the Kuroshio. Further research should focus on the nitrogen and carbon biogeochemical cycles of unicellular diazotrophs in the SCS and the Kuroshio. *Trichodesmium* presented to be a daytime N_2_ fixer with the maximum N_2_ fixation rate in the 8:00 to 10:00 am and the rate decreased sharply as time goes by. Free trichomes were the absolute dominant population in the study area, and the radial colony and the longitudinal colony were also observed during the survey time which could be caused by the shortage of nutrient or trace element. The average number of trichomes per colony in present study was lower than values reported in other oceans, and the contribution of *Trichodesmium* to total Chla presented spatial difference which might be caused by the variation of their dominant phytoplankton. N_2_ fixation by the whole-water in some stations contributed a lot to primary production in surface waters, suggesting that N_2_ fixation plays a very important role in supporting surface phytoplankton N demand in these areas.

## Materials and Methods

### Station locations and sampling

**Figure 8:**
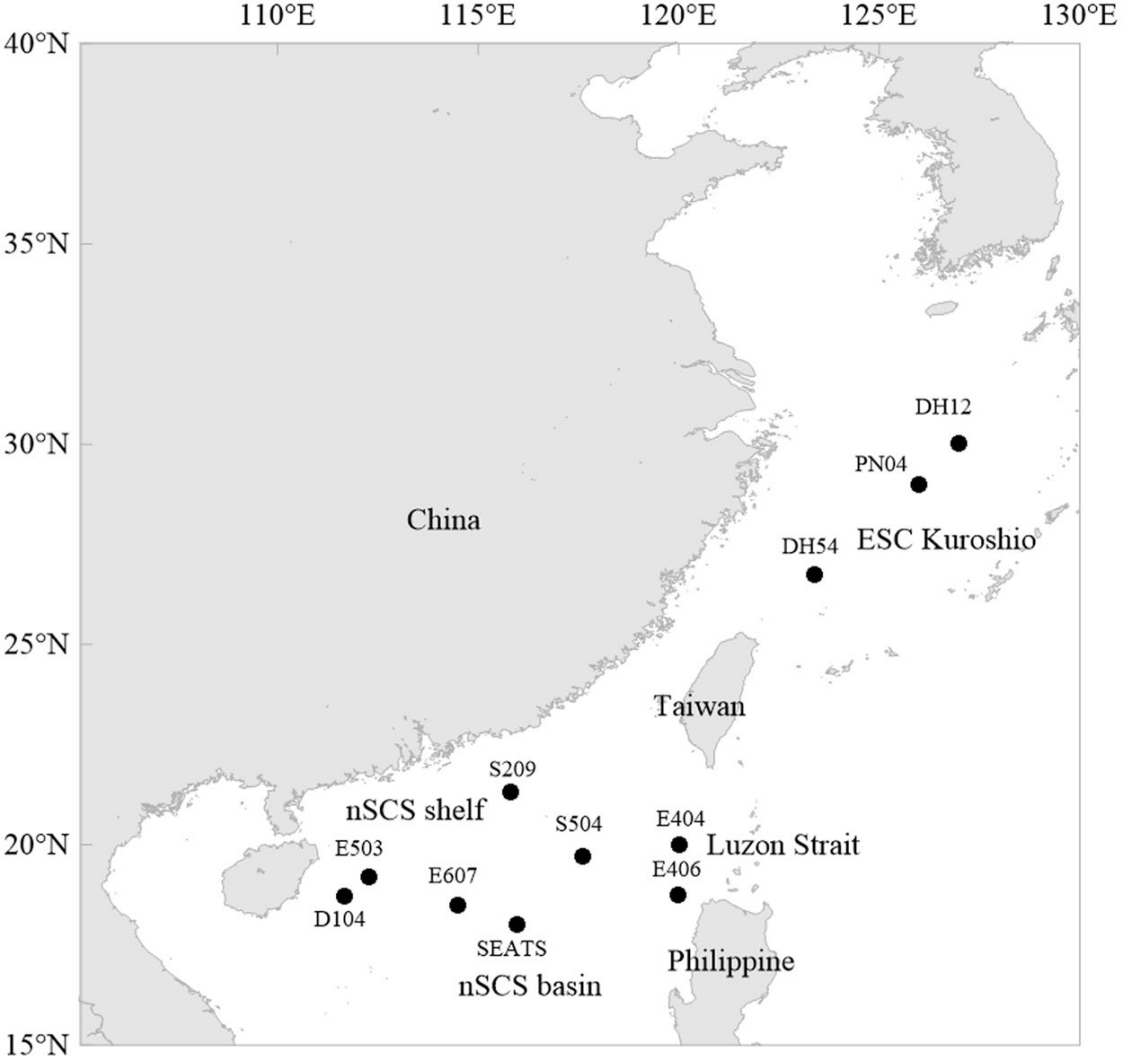
Sampling stations of N_2_ fixation rate. Note for software: Golden Software Surfer 11 (http://www.goldensoftware.com/).

The study area was located between 15°N to 35°N and 105°E to 130°E (Fig. 8). A total of 13 sampling stations were studied during the summer of 2009. Surface water was collected using an HCl-rinsed bucket at all the stations. Vertical seawater was collected by using 30 L Go-Flo bottles attached to a rosette multi-sampler, on which the conductivity, temperature, and depth (CTD) probes were installed (Seabird SBE 9/11). Temperature and salinity were profiled vertically by the CTD profiler.

### Nutrients and Chl *a* measurements

Water samples for determination of nutrients were collected into 100 ml HCl-rinsed bottles and stored in 4 °C until analysis. Soluble reactive phosphate (SRP) concentrations were measured using standard molybdenum blue procedure^57^ or a home-made ship-board C18 enrichment-flow injection analysis system^58,59^. Nitrate and nitrite concentrations (NO_3_^−^ + NO_2_^−^) were measured by reducing NO_3_^−^ to NO_2_^−^ with a Cd column and then determining NO_2_^−^ using the standard pink azo dye method and a flow injection analyzer^60^.

Chl *a* samples were collected in 1 L bottles and then vacuum filtered (<10 mm Hg) through a 25 mm GF/F filter. Filters were kept in 10 mL vials and pigments were extracted with 90% acetone for 24 h inside a freezer at 4 °C. Chlorophyll concentrations of the extraction were then determined based on the fluorescence technique using a Trilogy (CHL NA, Model # 046) fluorometer on board.

### *Trichodesmium* enumeration and its contribution to Chl *a*

Water samples for determining the vertical distribution of *Trichodesmium* were collected from 25 m, 50 m, 75 m, 100 m and 150 m depths in each sampling station. Specimens for enumeration of *Trichodesmium* were prepared on board by filtering a 1–10 L water samples onto a 20 μm polycarbonate membrane filter (25 mm in diameter). Concentrations of the samples were fixed with 1% neutralized formaldehyde and preserved in the dark until analysis. Trichomes and colonies on the entire filter were counted under an inverted microscope. In order to determine the contribution of *Trichodesmium* to the total Chl *a*, 30–50 *Trichodesmium* colonies were picked up and washed in the filtered sea water. Then the chlorophyll concentrations of the colonies were measured using fluorescence method as described above.

### Primary production

Primary production (PP) was conducted according to Parsons *et al*.^61^ by ^14^C incubation method^61^. Seawater samples were collected from surface (Table 1) or five depths of the euphotic layer (Table 2), then prescreened through 200 μm mesh and filled into 250 mL acid-cleaned polycarbonate carboy (Nalgene; USA). Each sample was inoculated with 10 μCi NaH^14^CO_3_ before incubation, and carboys which used for incubate subsurface samples were covered with the neutral density filter to simulate the *in situ* irradiance. Carboys were incubated in an on-deck incubator cooled by running water pumped up from a 5 m depth. After 4 h incubation, seawater samples were filtered through 25 mm diameter GF/F filters, and then the filters were placed in the aluminum foil bags and stored in the −20 °C freezer until analysis. In the laboratory, filters were fumed with HCl to remove residual inorganic carbon before they were moved into the scintillation vials, next the 10 mL scintillation cocktail (Ultima Gold; PerkinElmer; USA) were added. The activity of radioactive was counted using a Tri-Carb 2800TR liquid scintillation counter (Perkin-Elmer; USA).

### Size-fractioned N_2_ fixation rates measurements

N_2_ fixation activity was measured by acetylene reduction assay^3^. Sampling stations were grouped into four sectors according to their bottom depths and locations (Fig. 8): the nSCS shelf (Sta. S209, E503 and D104), the nSCS basin (Sta. E607 and SEATS), the ECS Kuroshio (Sta. DH54 and PN04) and the Luzon Strait (Sta. E404 and E406). All experimental incubations were run in triplicate. 500 ml of surface seawater samples were introduced into a 600 mL HCl-rinsed polycarbonate bottles. After sealing with a butyl rubber stopper, 20 mL of acetylene was injected by replacing the same volume of headspace. Seawater samples that passed through a 10 μm mesh were also prepared for incubation of the surface samples in the same manner as whole seawater samples. Samples were incubated for 24 h in an on-deck incubator cooled by running water pumped up from a 5 m depth. At Sta. SEATS, subsurface samples were also obtained from 100%, 50%, 10%, 3% and 1% light depths relative to that of the surface to determine N_2_ fixation at subsurface layers. The light intensity of the subsurface samples was adjusted to the *in situ* density using neutral density filters.

N_2_ fixation by *Trichodesmium* was also measured by acetylene reduction assay. A total of 6 sampling stations were occupied to determine the N_2_ fixation rate of *Trichodesmium* (Fig. 8). Two sites were located in the ECS Kuroshio (Sta. PN04 and DH12), one site in the nSCS shelf (Sta. S209), one site in the Luzon Strait (Sta. E404) and two sites in the nSCS basin (Sta. SEATS and S504). *Trichodesmium* colonies were collected by a 76 μm mesh phytoplankton net towing gently in the surface water. *Trichodesmium* colonies were manually picked immediately by using a plastic bacteriological transfer loop and placed into a glass container with GF/F filtered seawater. Approximately 30–50 colonies were transferred to a 25 ml glass vial with 15 ml filtered seawater. The vials were sealed with butyl rubber stoppers. After injection of 2 ml acetylene, vials containing *Trichodesmium* colonies were incubated for 4–6 h in the daytime. To evaluate N_2_ fixation of *Trichodesmium*, their activity at each station was calculated from their numerical abundance and their N_2_ fixation activity per trichome.

At the end of the incubation, the gas phase of the vials was sampled with a 100 μl gastight syringe (Agilent) and manually injected into a flame ionization gas chromatograph (GC6890N, Agilent). The temperatures of injector, detector and oven were set at 250 °C, 200 °C and 60 °C respectively. The carrier gas was helium at the highest purity available at a flow rate of 9 mlmin^−1^. The supply of H_2_ and air for the FID were 30 mlmin^−1^ and 300 mlmin^−1^, respectively. The column was 20m-long wide-bore fused silica (0.53 mm inner diameter) packed with Porapak U (Agilent). The ethylene production during the incubation was calculated as Capone^62^. The produced ethylene was converted to fixed nitrogen with a molar ratio of 4:1. After the assay, the incubated samples were fixed with 1% neutralized formaldehyde for the later enumeration of *Trichodesmium*.
